# Occurrence of the siphonophore *Muggiaea atlantica* in Scottish coastal waters: source or sink?

**DOI:** 10.1093/plankt/fbw082

**Published:** 2017-01-24

**Authors:** Michael Blackett, Cathy H. Lucas, Katherine Cook, Priscilla Licandro

**Affiliations:** 1 Ocean and Earth Science, National Oceanography CentreUniversity of Southampton Waterfront Campus, European Way, Southampton, SO14 3ZH, UK; 2 Sir Alister Hardy Foundation for Ocean Science, The Laboratory, Citadel Hill, Plymouth, PL1 2PB, UK; 3 Marine Scotland Science, Marine Laboratory, 375 Victoria Road, Aberdeen Ab11 9DB, UK

**Keywords:** siphonophore, jellyfish, range expansion, source–sink dynamics, source, sink, biogeography, gelatinous zooplankton, bloom

## Abstract

We applied the concept of source–sink dynamics to investigate a recent (1999–2013) increase in the occurrence of the siphonophore *Muggiaea atlantica* in Scottish coastal waters. Our aim was to determine whether this change represented the establishment of resident populations (i.e. “sources”), or transient populations reliant on immigration (i.e. “sinks”). First, we show that local production was not always sufficient to account for recruitment (a “source” prerequisite), suggesting reliance on immigration (a “sink” prerequisite). Using variation partitioning, we then discriminated between the exclusive effects of immigration [indexed by the European Slope Current (ESC)] and local production (indexed by local sea temperature and food availability). On the west coast (Loch Ewe), interannual variability in the species’ abundance was determined by, in order of increasing importance: (i) suitable local environmental conditions (13%); (ii) the role of the ESC in modulating these conditions (20%); and (iii) immigration *via* the ESC (29%). These results provided a strong indication that Loch Ewe represents a sink habitat for *M. atlantica*. However, on the east coast (Stonehaven) our results were less conclusive, probably due to the less direct influence of the ESC. For both locations, we suggest that low winter temperatures prevented overwintering, necessitating annual re-colonization *via* immigration.

## INTRODUCTION

Jellyfish (Cnidaria and Ctenophora) represent an important functional group of secondary consumers in marine pelagic ecosystems (Condon *et al*., 2012; Lucas *et al*., 2014). The ecological interactions of jellyfish can modify ecosystem structure and function (Mills, 1995; Pitt *et al*., 2009), with knock-on effects for a range of ecosystem services (Purcell, 2012). Over recent decades, increases in the abundance and distribution of jellyfish have been reported in many regions of the world (Purcell *et al*., 2007; Brotz *et al*., 2012). Global climate change is modifying the marine environment, resulting in changes to the spatial and temporal distribution of marine species (Edwards and Richardson, 2004; Beaugrand *et al*., 2014). In the context of a changing global climate, it is important to develop understanding of the factors and mechanisms that drive shifts in populations of jellyfish. A critical aspect of this understanding is our capacity to distinguish the effects of both biological (e.g. changes to demographic rates) and physical (e.g. changes to immigration and emigration rates) processes on jellyfish populations (Graham *et al*., 2001).

The concept of source–sink dynamics provides a theoretical framework to help understand how dispersal and habitat-specific demography influence species abundance and distribution (Pulliam, 2000). In source habitats, local production exceeds local mortality, and emigration exceeds immigration (dispersal), whereas in sink habitats, the opposite is true. Source habitats are, by definition, self-sustaining; they provide the necessary conditions for a species to complete its full life history and to persist indefinitely, independent of the contribution of immigration. Conversely, unsuitable conditions in sink habitats prevent the species from persisting indefinitely and populations must rely upon immigration to balance losses to local mortality (and emigration). The result is that, in spatially complex landscapes, habitat heterogeneity can generate spatially explicit population structures composed of networks of source and sink habitats all connected *via* dispersal (Dunning *et al*., 1992). Recent studies have demonstrated the importance of source–sink dynamics in the modulation of the spatial and temporal distribution of jellyfish populations (Costello *et al*., 2012; Chen *et al*., 2014).

The cnidarian jellyfish *Muggiaea atlantica* (Siphonophorae, Calycophora) is an important gelatinous predator in low- to mid-latitudinal coastal waters of the three major oceans (Mapstone, 2014). During the late 20th century, expansion of the species’ distribution has been reported in a number of different regions including the Mediterranean (Licandro *et al*., 2012; Batistić *et al*., 2013) and South Pacific (Palma *et al*., 2014). Intense predation by *M. atlantica* can deplete prey resources, restricting the available energy for other functional groups and disrupting the balance of the ecosystem (Greve, 1994; Kršinić and Njire, 2001). As a vector for disease (Fringuelli *et al*., 2012), and by injuring and killing farmed fish (Baxter *et al*., 2011), *M. atlantica* is also capable of inflicting significant economic losses on aquaculture operations (Fosså *et al*., 2003; Cronin *et al*., 2004). To predict and manage these impacts, it is important to better understand the processes that influence the population dynamics and spatio-temporal patterns of *M. atlantica* in different regions.

Throughout the 20th century, *M. atlantica* was almost entirely absent from the Scottish Continental Shelf (Fraser, 1967; Heath *et al*., 1999). However, more recent observations suggest that the species’ frequency of occurrence has dramatically increased (K. Cook, Aberdeen, personal observation). We previously demonstrated the transition of the Western English Channel (WEC) from a sink to a source habitat for *M. atlantica* in the late 1960s (Blackett *et al*., 2014). As reported for jellyfish in other regions (Bolte *et al*., 2013), the formation of this source may have augmented the species’ dispersal range, enabling the formation of new populations in the previously uninhabited Scottish region. In jellyfish, source and sink habitats are connected *via* current-driven transport (Costello *et al*., 2012; Chen *et al*., 2014). The European Slope Current (ESC) flows along the northwest European Continental Shelf from the Bay of Biscay to the Faroe-Shetland Channel (Xu *et al*., 2015); and the presence of *M. atlantica* in the Scottish Continental Shelf has been associated with the inflow of these waters (Fraser, 1967). An investigation into the role of source–sink dynamics would aid our understanding of the apparent northward biogeographical expansion of *M. atlantica* in the northeast Atlantic Ocean.

In this study, we used time series data from the source habitat of the WEC and two potential habitats on the east and west coasts of Scotland to investigate how source–sink processes influence temporal patterns of *M. atlantica* abundance in Scottish waters. At the Scottish sampling locations, we identified local production and the influence of key environmental parameters chosen on the basis of published studies. Then, using a form of variation partitioning we examined the respective contribution of local production (as indexed by the key local environmental parameters) and immigration (as indexed by the ESC and source abundance) on the annual abundance of *M. atlantica* in Scottish coastal waters. The aims of this study were (i) to confirm the species increased frequency of occurrence in Scottish waters; (ii) to assess the status of Scottish waters as either sources or sinks; and (iii) to determine the role of the WEC as a source of *M. atlantica* propagules for immigration into Scottish waters. Through these efforts, we attempt to add to our understanding of the mechanisms that drive changes in the biogeography of *M. atlantica* in the northeast Atlantic Ocean and adjacent seas.

## METHOD

### Muggiaea data

**Fig. 1.**
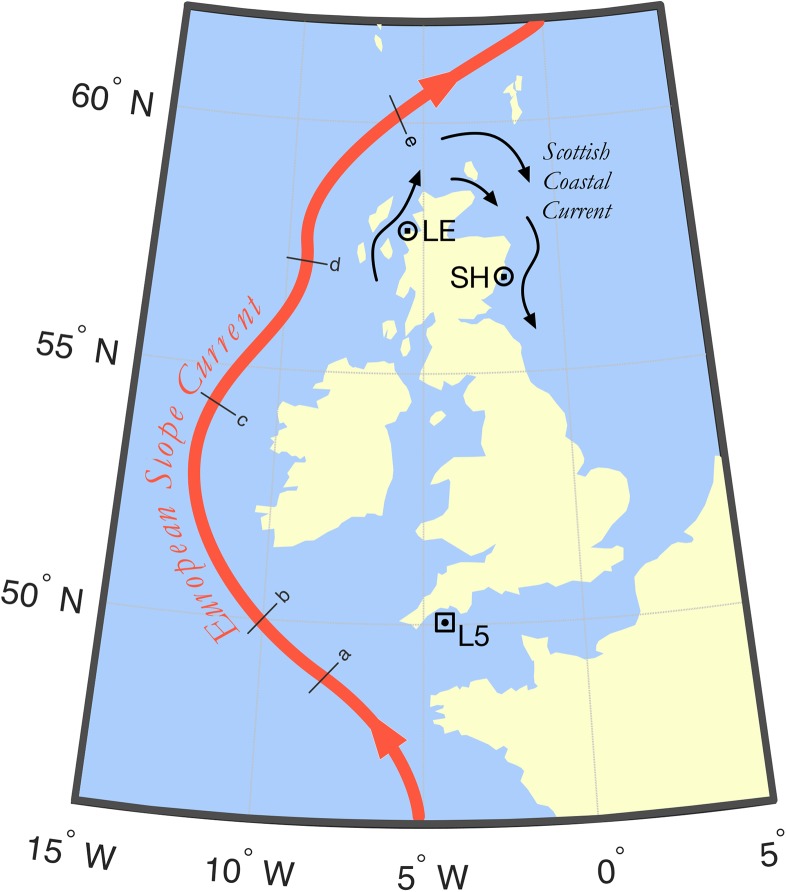
Map of the British Isles with the sampling locations and the general patterns of water circulation indicated. LE, Loch Ewe; SH, Stonehaven; L5, Western English Channel shelf station. The European Slope Current sampling sections are indicated: (**a**) Celtic; (**b**) Goban Spur; (**c**) Irish; (**d**) Hebridean; (e) Shetland.

Data on the abundance of *Muggiaea atlantica* (polygastric and eudoxid stages only) in Scottish coastal waters were obtained from two coastal monitoring stations operated by Marine Science Scotland: (i) Loch Ewe, a sea loch in the Northwest Highlands of Scotland and (ii) Stonehaven, a North Sea station ~5 km off the coast of Aberdeen (Fig. 1 and Table I). Data from the WEC (polygastric stage only) were derived from the open-shelf time series records provided by the Western Channel Observatory (Table I) and previously described by Blackett *et al*. (2014).

**Table I:** Sources and characteristics of plankton and environmental data used in this study

Location	Data	Units	Coverage	Frequency	Methodology	Source
Loch Ewe	Polygastric	Col. m^−3^		Weekly	Vertical hauls ø = 0.4 m Mesh = 200 μm	MSS
57.85°N						
5.65°W	Eudoxid		2003–2013			
40 m depth						
	Food availability	Ind. m^−3^				

	Sea temperature (1–5 m and 30–35 m)	°C	2003–2013	Weekly	Reversing thermometer	

	Salinity (1–5 m and 30–35 m)				Salinometer	

Stonehaven	Food availability	Ind. m^−3^	1999–2013	Weekly	Vertical hauls ø = 0.4 m Mesh = 200 μm	MSS
56.96°N						
02.10°W	Polygastric	Col. m^−3^	1999–2013			
50 m depth						
	Eudoxid		2001–2006			

	Sea temperature (1 m and 40–48 m)	°C	1999–2013	Weekly	Reversing thermometer	

	Salinity (1 m and 40–48 m)				Salinometer	

WEC L5	Polygastric	Col. m^−3^	2003–2012	Monthly	Double-oblique hauls *A* = 0.4 m^2^ Mesh = 700 μm	WCO
50.03°N						
04.37°W						
65 m depth						
NW European shelf	Slope current	cm s^−1^	1999–2012	Weekly	Satellite altimetry/*in situ* observations	Xu*et al*. (2015)

MSS, Marine Science Scotland; WCO, Western Channel Observatory; ø: Diameter; *A*, Area; Col., Colonies; Ind., Individuals. Food availability was represented by the abundance of copepods.

Details of the different sampling protocols are summarized in Table I. For all data sets, the number of *M. atlantica* nectophores was used as a direct estimate of the abundance of the polygastric stage, as species of the genus *Muggiaea* develop only a single nectophore (Mackie *et al*., 1987). The eudoxid stage of *M. atlantica* was enumerated using the number of detached bracts and intact colonies. Whilst the eudoxid stages of the genus *Muggiaea* are morphologically indistinguishable (Kirkpatrick and Pugh, 1984), eudoxid identity was attributed to *M. atlantica* because it was the only species of *Muggiaea* recorded at the Scottish sampling locations.

### Local environmental data

Sea temperature and salinity (at surface and bottom depths), and food availability were recorded simultaneously with *Muggiaea* data at the Scottish sampling locations (Table I). Temperature and food availability were essential parameters to consider due to their proven effects on reproduction in *Muggiaea* (Purcell, 1982; Carré and Carré, 1991). Whilst *M. atlantica* displays euryhaline characteristics (Palma *et al*., 2014), salinity was still an important variable to consider due to its utility as a water mass indicator (Blackett *et al*., 2014). Total copepod abundance (excluding species with <25% frequency of occurrence) was used as an index of food availability as they represent the main dietary component for *M. atlantica* (Purcell, 1981, 1982).

### ESC data

The ESC is a major section of the poleward flow of Atlantic water into the North Sea and Nordic Seas (Xu *et al*., 2015). We used the ESC as an index of the potential immigration of *M. atlantica* into the Scottish sampling locations. Interannual variability in the magnitude of the ESC was assessed using the 20-year time series constructed by Xu *et al*. (2015). Weekly data (1999–2012) from a series of sampling sections [extending from the WEC to the Hebrides (Loch Ewe) or the Shetland Channel (Stonehaven)] were used to calculate the annual ESC time series (Fig. 1). For a detailed description of the methodology, see Xu *et al*. (2015).

### Numerical analysis

All data manipulation and statistical analysis was conducted in the MATLAB1 environment (R2015b 8.6.0.267246).

### Data preparation

Random missing values (<10% of the total observations) in the weekly plankton abundance and local environmental data were estimated using shape-preserving piecewise cubic interpolation. The sampling frequency was then adjusted to a monthly frequency. To stabilize variance, the monthly plankton time series were transformed [log_10_ (*x* + 1)]. The next step was to calculate annual time series for all of the variables. For the Scottish sampling locations, we focussed on the seasonal period during which *M. atlantica* was present; the annual scores were calculated as the arithmetic mean [i.e. using the back-transformed (anti-log) plankton data] of the consecutive months displaying a greater than 25% frequency of occurrence of *M. atlantica*. For the WEC location, the annual *M. atlantica* time series was calculated using all 12 months, as the species is typically present throughout the year (Blackett *et al*., 2014, 2015). Annual scores for the plankton time series were then transformed [log_10_ (*x* + 1)] to stabilize variance before all annual variables were standardized to zero mean and unit deviation (*z*-scored). Finally, the presence of any significant interannual trends was tested using up and down runs tests (Legendre and Legendre, 2012). As no significant temporal trends were identified in the annual time series, further analyses were conducted on the standardized annual anomalies.

### Identification of local *M. atlantica* production

We identified periods of local biological production using the technique described by Blackett *et al*. (2014). This procedure involved the identification of the quantity of information associated with peaks and troughs (turning points) in the monthly time series of *M. atlantica* abundance. Turning points associated with a high quantity of information represent the culmination of gradual changes in abundance, whereas those turning points associated with a low quantity of information reflect abrupt (random) fluctuations. We considered the consecutive months between troughs and peaks associated with a significant high quantity of information (>4.2 bits) (Ibañez, 1982) as periods of population increase characteristic of local biological production.

### Local environmental influence at the seasonal scale

To verify the influence of local environmental conditions on abundance, we identified significant associations with each of the local environmental parameters (sea temperature, salinity and food availability). Associations were identified using quotient analysis (Van Der Lingen *et al*., 2001). Abundance quotients were calculated as the percentage of the total abundance recorded within categories of local environmental parameters, divided by the percentage frequency of occurrence of each of the categories. Quotient values above 1 indicate a positive association of abundance with a specific environmental category, while quotients values below 1 indicate a negative association and quotient values of 1 indicate random association (Van Der Lingen *et al*., 2001). In the present study, the quotient values were log transformed (to the base 2) to aid graphical representation of the negative quotients (that range only between 0 and 1); this transformation resulted in the test criterion diverging from 0 (instead of 1). Only the months when *M. atlantica* was present were considered. To test the null hypothesis of associations occurring merely by chance, a permutation test with 10 000 repetitions was used to calculate confidence intervals with *α* = 0.05.

### Source–sink processes at the annual scale

We used partial linear regression (Peres-Neto *et al*., 2006) to assess the respective contribution of endogenous (e.g. local demographic rates) and exogenous (e.g. immigration and emigration rates) processes to the interannual variability in the abundance of *M. atlantica*. This method of variation partitioning allows estimation of the amount of variation in a response variable that can be attributed exclusively to one set of explanatory variables, once the effect of the other set has been taken into account and controlled for (Legendre and Legendre, 2012). This technique was used to test the null hypothesis that the Scottish sampling locations represent a source habitat, against the alternative hypothesis that they represent a sink habitat. In a source habitat, local production outweighs immigration (Pulliam, 2000) and therefore effects of local environmental variability on the species intrinsic (e.g. reproduction, fecundity, mortality) dynamics would be expected to explain the majority of the variability in abundance. Conversely, in a sink habitat, immigration outweighs local production (Pulliam, 2000) and therefore translocation would be expected to explain the majority of the variability. The steps of the method are briefly described below, following Legendre and Legendre (2012).

**Fig. 2.**
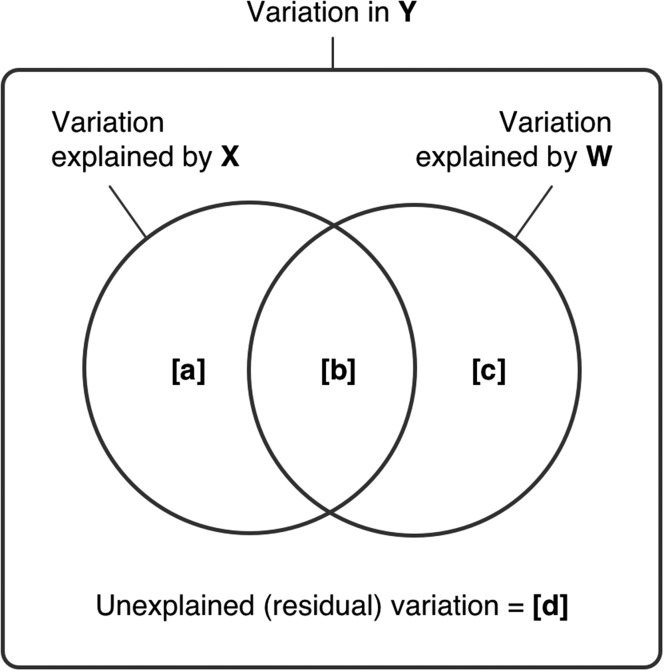
Schematic Venn diagram representing the partition of variation of a response variable, **y**, among two sets of explanatory variables, **X** and **W**. The outer rectangle represents 100% of the variation in y. Fractions [a] and [c] represent the portion of variation attributed exclusively to **X** and **W**, respectively, while fraction [b] represents the intersection (not the interaction) of the variation explained by both **X** and **W**. The fraction [a + b + c] represents the total variability explained by both**X** and **W** together, while fractions [a + b] and [b + c] represent the exclusive and common variation explained by **X** and **W**, respectively. Adapted from Legendre and Legendre (2012).

Let **y** represent the response variable (*M. atlantica* abundance), **X** one set of explanatory variables (local environmental factors: sea temperature, salinity and food availability) and **W** (also called “the matrix of covariables”) the other set of explanatory variables (translocation factors: the ESC and source abundance). First, the multiple linear regression of **y** against **X** and **W** together is computed. The corresponding coefficient of multiple determination (*R*^2^) represents the fraction of the variation in **y** explained by both **X** and **W** (i.e. fraction [a + b + c], [a + b], [b + c], [a], [b], [c] and [d] defined in Fig. 2). Next, the multiple linear regression of **y** against **X** is computed, with the corresponding *R*^2^ representing the fraction of variation explained by **X** (i.e. fraction [a + b]; Fig. 2). Similarly, the multiple linear regression of **y** against **W** is then computed, with its corresponding *R*^2^ representing the fraction of variation explained by **W** (i.e. fraction [b + c]; Fig. 2). Finally, the individual fractions [a], [b], [c] and the (residual) fraction [d] are calculated by subtraction. These individual fractions represent:

[a] The variation explained by **X** (local environmental factors) once the effect of **W** (translocation factors) has been removed;
[b] The common variation explained by both **X** and **W** (their intersection *not* interaction);
[c] The variation explained by **W** once the effect of **X** (local environmental factors) has been removed;
[d] The unexplained (residual) variation.

We considered the translocation factors as the matrix of covariables because the ESC influences both plankton dispersal and the local environmental conditions (Beaugrand *et al*., 2001; Inall *et al*., 2009). The local environmental variables used to populate set **X** were chosen following demonstration of their influence on local production. A stepwise selection procedure (with a probability-of-F-to-enter ≤0.05 and two-tailed *P*-values) was then applied separately to the sets of explanatory variables, **X** and **W**. Provided the conditions of homoscedasticity, independence and normality of the residuals are satisfied, the significance of the fractions [a + b + c], [a + b], [b + c], [a] and [c] can be tested under the parametric framework; otherwise, *P-*values can be obtained using a permutation test (Legendre and Legendre, 2012). Fraction [b] is not an interaction term (as in the ANOVA family of analyses) and cannot be tested for statistical significance. Multicollinearity was evaluated using Belsley collinearity diagnostics (Belsley *et al*., 1980). The adjusted coefficient of multiple determination ($R_{a}^{2}$) was used here because **X** and **W** contained random variables (Peres-Neto *et al*., 2006).

## RESULTS

### Loch Ewe: seasonal variability and local *M. atlantica* production

**Fig. 3.**
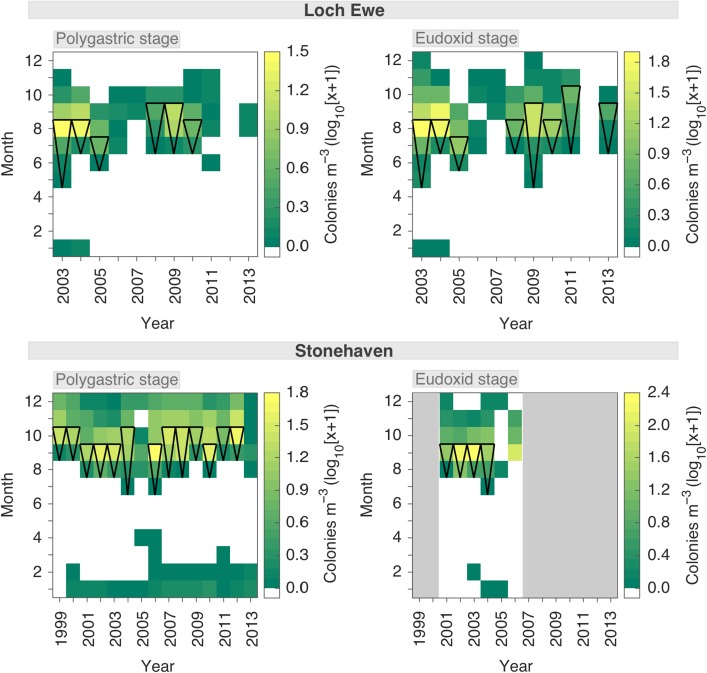
Patterns of seasonal variability in the abundance of *Muggiaea atlantica* in Scottish coastal waters. Abundance (log_10_ [colonies m^−3^ + 1]) of the polygastric and eudoxid stages at Loch Ewe (west coast) between 2003 and 2013 (top panel), and at Stonehaven (east coast) between 1999 and 2013 (bottom panel). Periods of population increase characteristic of local biological production are represented by black triangles.

The seasonal occurrence of *M. atlantica* at Loch Ewe between 2003 and 2013 was ephemeral (Table II), being present in only 33–39% of the monthly observations. The species was generally absent throughout the winter and much of the spring, with both stages typically appearing in early summer and remaining present until early autumn (Fig. 3). On average, the seasonal period of maximum abundance was from May to November. During 2006 and 2007, the seasonal distribution of *M. atlantica* was particularly restricted. This pattern was also apparent in 2013 for the polygastric stage. In 2012, both stages were absent from the Loch. The monthly abundance of the two stages significantly covaried (*r* = 0.94, *P* < 0.001), with the eudoxid stage typically attaining greater densities (Table II).

**Table II:** Mean, standard deviation, maximum abundance (colonies m^−3^) and percentage of zero values in the monthly time series of *Muggiaea atlantica* abundance at the Scottish sampling locations: Loch Ewe (2003–2013) and Stonehaven (1999–2013)

	Mean	SD	Max	Zeros (%)
Loch Ewe				
Polygastric	0.9	3.7	3.4	67
Eudoxid	2.8	10.5	13.1	61
Stonehaven				
Polygastric	3.6	9.1	23.3	46
Eudoxid	8.5	32.7	33.1	63

SD, standard deviation; Max: 95th centile.

During the periods 2003–2005 and 2008–2010, local population development commenced immediately following the species appearance in the loch. This production progressed for 2- to 4-month periods before culminating in peak population density in August–September (Fig. 3). During the years characterized by a restricted period of occurrence (2006 and 2007), local population development was not recorded. Both stages typically displayed similar patterns of population development; however, the eudoxid stage appeared and commenced development earlier than the polygastric stage in 2009 and it also peaked earlier in 2008. In 2011 and 2013, only the eudoxid stage was developing *in situ*.

### Loch Ewe: local environmental optima at the seasonal scale

The results of the quotient analysis were virtually identical for the polygastric and eudoxid stages of *M. atlantica* at Loch Ewe; and therefore, the species associations are described here in terms of the polygastric stage only.

**Fig. 4.**
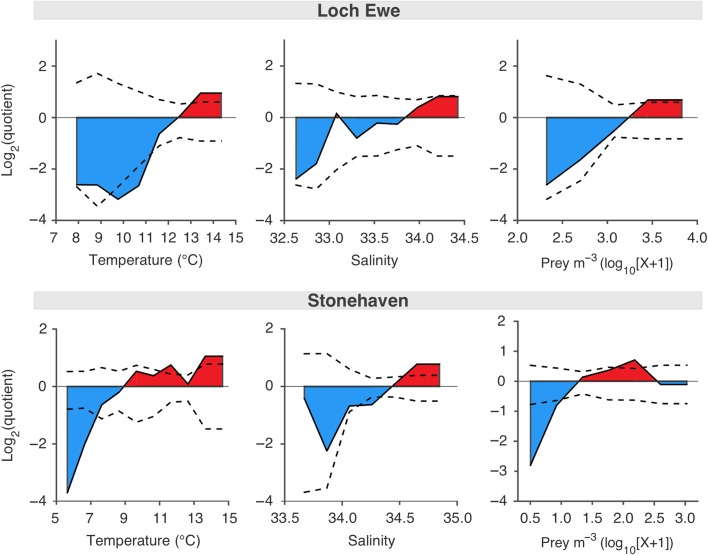
Preferential ranges and critical thresholds of local environmental conditions associated with the abundance of *Muggiaea atlantica* in Scottish coastal waters. Associations of polygastric stage abundance with SST, salinity and food availability (copepod prey abundance m^−3^) at Loch Ewe (east coast) between 2003 and 2013 (top panel), and at Stonehaven (west coast) between 1999 and 2013 (bottom panel). Quotient values above 1 indicate a positive association (in red), while quotient values below 0 indicate a negative association (in blue) and quotient values of 0 indicate a random association. The dotted lines delimit 95% confidence intervals.

Abundance was positively related to SST. Occurring within the range of 8.0–14.4°C, high abundance of *M. atlantica* was significantly (*P* < 0.05) associated with SST between 12.8°C and 14.4°C, while a significant negative association was identified with SST in the range of 9.5–11.2°C (Fig. 4). *Muggiaea atlantica* was not recorded in the lowest SST range of 6.3–7.9°C. The abundance of *M. atlantica* was positively related to salinity; however, this association was not statistically significant (Fig. 4). The species was not recorded when the salinity was low (<32.60). The species’ associations with the temperature and salinity at bottom depth were consistent with those observed at the surface (data not shown). High abundance of *M. atlantica* was significantly associated with the maximum abundance of copepods; however, there was no significant negative association with low food availability (Fig. 4).

### Loch Ewe: interannual variability

**Fig. 5.**
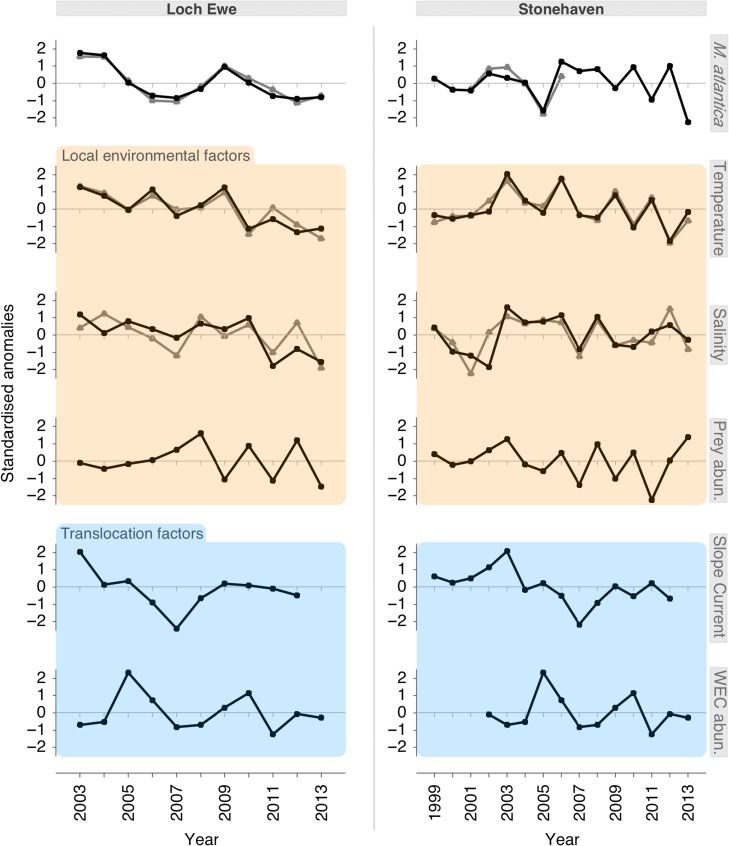
Interannual variability in the abundance of Muggiaea atlantica in Scottish coastal waters, together with local environmental factors and translocation factors. Left panel: Loch Ewe (west coast); Right panel: Stonehaven (east coast). The abundance of M. atlantica is represented by both the polygastric (black lines) and eudoxid stages (grey lines). Local environmental factors include: sea temperature at surface (black line) and bottom (grey line); salinity at surface (black line) and bottom (grey line); the abundance of copepod prey. Translocation factors include: the strength of the European Slope Current; the abundance of M. atlantica (polygastric stage) in the source habitat of the Western English Channel (WEC). Values are represented by standardised annual anomalies; annual anomalies for the Loch Ewe and Stonehaven variables were calculated using the seasonal period of M. atlantica occurrence (May–November and August–December, respectively).

Between 2003 and 2013, the long-term mean annual abundance (±standard deviation) of *M. atlantica* at Loch Ewe was 1.37 ± 1.98 and 4.27 ± 5.62 colonies m^−3^ for the polygastric and eudoxid stages, respectively. The abundance of the two stages was highly correlated (Table III). Interannual changes in population density displayed a quasi-bimodal distribution characterized by two periods of high abundance in 2003–2004 and 2009 flanked by periods of low abundance (Fig. 5).

**Table III:** Correlation table of *Muggiaea atlantica* and environmental parameters utilized in the Loch Ewe variation partitioning analysis.

Variable	PG_LE_	EX_LE_	SST_LE_	SBT_LE_	SS_LE_	BS_LE_	FA_LE_	ESC	PG_WEC_
PG_LE_	1.00								
EX_LE_	**0.98**	1.00							
SST_LE_	**0.63**	**0.55**	1.00						
SBT_LE_	0.60	0.52	**0.93**	1.00					
SS_LE_	0.52	0.48	0.43	0.17	1.00				
BS_LE_	0.46	0.45	0.08	−0.05	0.50	1.00			
FA_LE_	−0.40	−0.45	−0.47	−0.57	0.26	0.38	1.00		
ESC	**0.74**	**0.77**	0.36	0.33	0.37	0.44	−0.36	1.00	
PG_WEC_	−0.04	−0.01	−0.06	−0.28	0.48	0.25	0.03	0.13	1.00

Pearson's correlation coefficients were computed between the standardized annual anomalies (2003–2013). To account for temporal autocorrelation *P-*values were calculated using the adjusted degrees of freedom (Pyper and Peterman, 1998).

PG, polygastric stage; EX, eudoxid stage; SBT, sea bottom temperature; SS, surface salinity; BS, bottom salinity; FA, food availability; LE, Loch Ewe; bold: significant relationship (*P* < 0.05).

#### Local environmental factors

The long-term annual (May–November) means and standard deviations of local SST and salinity were 11.45 ± 0.36°C and 33.44 ± 0.49, respectively. Concomitant with changes in the local abundance of *M. atlantica*, SST and salinity were maximal in 2003 and 2009/2010 and minimal in 2007 and after 2010 (Fig. 5). Temperature and salinity variability at bottom depth were coupled with changes at the surface (Fig. 5). The annual (May–November) mean abundance of copepod prey (2882 ± 1044 ind. m^−3^) gradually increased from 2003 until 2008, after which it fluctuated above and below the long-term mean (Fig. 5). Overall, only sea temperature was significantly positively related to the local abundance of *M. atlantica* (Table III).

#### Translocation factors

The long-term annual mean ESC velocity and its standard deviation were 0.35 ± 0.92 cm s^−1^. Interannual variability in the magnitude of the ESC was significantly positively correlated with the population density of *M. atlantica* at Loch Ewe (Table III), revealing a remarkable level of synchronicity (Fig. 5; Table III). The long-term mean annual abundance of *M. atlantica* and its standard deviation in the open-shelf waters of the WEC (station L5) was 1.37 ± 0.93 polygastric colonies m^−3^. The population density of *M. atlantica* in Loch Ewe was not significantly linked to the species abundance in the WEC (Fig. 5; Table III).

### Loch Ewe: source–sink dynamics at the annual scale

The results of the variation partitioning analysis were similar for both the polygastric and the eudoxid stages of *M. atlantica*; the results are described here in terms of the polygastric stage only (see Table IV for the eudoxid stage results).

**Table IV:** Results of variation partitioning of interannual (2003–2013) variability in the abundance of the Loch Ewe *M. atlantica* polygastric stage among the local environmental (SST) and translocation (ESC) factors

Fraction of variation	Explanatory variables	$R_{a}^{2}$	d.f.	*F*	*β*
[a + b + c]	SST	0.62 (0.60)	7	**8.23 (7.64)**	0.44 (0.33)
	ESC				**0.53 (0.59)**
[a + b]	SST	0.33 (0.22)	8	**5.34 (4.55)**	**0.63 (0.58)**
[b + c]	ESC	0.49 (0.55)	8	**9.60 (11.80)**	**0.67 (0.70)**
[a]	SST	0.13 (0.05)			
[b]	*Intersect*	0.20 (0.17)			
[c]	ESC	**0.29 (0.38)**			
[d]	*Residual*	0.38 (0.40)			

Values in parentheses correspond to the eudoxid stage. Fraction [a + b + c] represents the variation explained by both sets of explanatory factors, fraction [a + b] the variation explained by SST and fraction [b + c] the variation explained by the ESC. Fractions [a] and [c] represent the portion of variation attributed exclusively to SST and the ESC, respectively, while fraction [b] represents their intersection (not interaction) and [d] the residual (unexplained) variation.

Bold: statistical significance (*P* < 0.05).

A stepwise selection procedure selected (*α* < 0.05) SST as the local environmental factor (set **X**) and the ESC as the translocation factor (set **W**). The residuals derived from the regression models were not normally distributed; and therefore, the significance of the fractions of variation was tested using a permutation test with 10 000 repetitions (Legendre and Legendre, 2012).

**Fig. 6.**
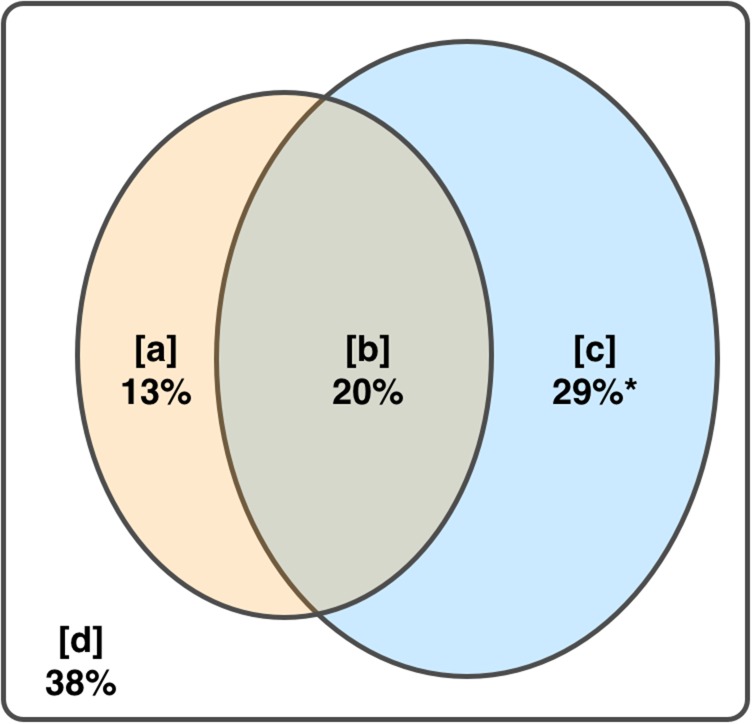
Proportional Venn diagram representing the fractions of variation of the annual abundance of the *Muggiaea atlantica* polygastric stage at Loch Ewe (2003–2012) attributed exclusively to: [**a**] the local environmental factor (SST); [**c**] the translocation factor (strength of the ESC); [**b**] the common variation explained by both factors; and [**d**] the residual (unexplained) variation. Numbers represent the percentage of the total variability. ^*^P < 0.01.

Together, the local environmental factor and the translocation factor (i.e. SST and the ESC, respectively) (fraction [a + b + c]) explained a large proportion of the total variation in the annual abundance (~62%; Table IV). Alone, the local environmental factor (SST; fraction [a + b]) accounted for ~33% of the total variation, while the translocation factor (the ESC; fraction [b + c]) accounted for 49% (Table IV). However, these two fractions contain the common variation explained by one another (fraction [b]). When this common variation was removed, only ~13% of the total variation explained by fraction [a + b] was attributed exclusively to SST (fraction [a]). In contrast, over half of the variation associated with fraction [b + c] (~29% of the total variation) was attributed exclusively to the ESC (fraction [c]) (Fig. 6).

### Stonehaven: seasonal variability and local *M. atlantica* production

At Stonehaven (1999–2013), the monthly frequency of occurrence of *M. atlantica* (54%) was greater than observed at Loch Ewe (Table II). However, the species was still absent throughout most of spring and summer. The period of maximum population density was from August to December, revealing a delayed phenology compared with Loch Ewe (Fig. 3). In 2005 and 2013, the occurrence of the polygastric stage was more sporadic than observed during the other years. During the period of coincident data coverage (2001–2006), the monthly abundance of the polygastric stage and the eudoxid stage significantly covaried (*r* = 0.92, *P* < 0.001), with the eudoxid stage typically more abundant (Table II).

The polygastric stage of *M. atlantica* displayed characteristic local population development during all of the years analysed except 2005 and 2013 (Fig. 3). Available data showed that population development of the eudoxid stage matched that of the polygastric stage, except in 2006. As was observed at Loch Ewe, population development commenced with the appearance of the stages. Population growth progressed for 2–4 months, reaching peak densities in September–October. In general, *M. atlantica* appeared ~2 months later, and peaked 1 month later, than observed at Loch Ewe. However, at Stonehaven the species attained greater annual abundance (Table II), and the patterns of occurrence and population development were more regular.

### Stonehaven: local environmental optima at the seasonal scale

Due to the limited number of observations on the abundance of the eudoxid stage of *M. atlantica* at Stonehaven, quotient analysis was only performed on the polygastric stage data.

The abundance of the polygastric stage was positively related to SST. Occurring within the range of 5.6–14.7°C, high abundance of *M. atlantica* was significantly (*P* < 0.05) associated with the maximum temperature range of 13.1–14.7°C, while a significant negative association was identified with the temperature range of 5.6–7.4°C (Fig. 5). *Muggiaea atlantica* was not recorded at the minimum SST range (4.9–5.6°C). The polygastric stage also displayed a positive relationship with salinity (Fig. 5). Occurring in the range 33.70–34.85, high abundance of the polygastric stage was significantly associated with the maximal recorded salinities (34.50–34.85). There was no consistent negative association with salinity. Abundance of the polygastric stage was significantly positively associated with high food availability and significantly negatively associated with low food availability (Fig. 5). The species’ associations with the temperature and salinity at bottom depth were consistent with those observed at the surface (data not shown).

### Stonehaven: interannual variability

At Stonehaven, the long-term (1999–2013) mean annual abundance (±standard deviation) of the polygastric stage of *M. atlantica* was 8.49 ± 5.57 colonies m^−3^. For the eudoxid stage, it was 20.42 ± 17.60 (2001–2006). When data on both the polygastric and eudoxid stages were available, the abundance of the two stages covaried (Fig. 5). The pattern of interannual variability in abundance involved a relatively stable period from 1999 to 2004, with abundance around the long-term mean. In 2005, the abundance was particularly low, while from 2006 to 2008 it was stable above the long-term mean. From 2009 onwards, the annual abundance of *M. atlantica* fluctuated above and below the mean, with the minimum abundance recorded in 2013 (Fig. 5).

#### Local environmental factors

The long-term annual (August–December) means and standard deviations of local SST and salinity were 11.71 ± 0.40°C and 34.52 ± 0.13, respectively. Temperature and salinity at bottom depth were closely linked to the variability observed at the surface (Fig. 5). Neither of these parameters displayed any significant relationship with *M. atlantica* (Table V and Fig. 5). The long-term mean abundance of copepods and its standard deviation were 193.6 ± 93.6 ind. m^−3^; changes in the local abundance of *M. atlantica* were significantly positively related to this measure of food availability (Table V and Fig. 5).

**Table V:** Correlation table of *Muggiaea atlantica* and environmental parameters utilized in the Stonehaven variation partitioning analysis

Variable	PG_SH_	SST_SH_	SBT_SH_	SS_SH_	BS_SH_	FA_SH_	ESC	PG_WEC_
PG_SH_	1.00							
SST_SH_	−0.08	1.00						
SBT_SH_	−0.16	**0.96**	1.00					
SSS_SH_	0.07	0.40	0.23	1.00				
SBS_SH_	0.24	0.10	0.03	**0.73**	1.00			
FA_SH_	**0.53**	0.06	−0.03	0.25	0.44	1.00		
ESC	−0.34	0.42	0.41	0.07	0.16	0.36	1.00	
PG_WEC_	−0.24	−0.15	−0.04	−0.01	0.19	0.15	0.02	1.00

Pearson's correlation coefficients were computed between the standardized annual anomalies (1999–2013). To account for temporal autocorrelation, *P-*values were calculated using the adjusted degrees of freedom (Pyper and Peterman, 1998).

PG, polygastric stage; SBT, sea bottom temperature; SS, surface salinity; BS, bottom salinity; FA, food availability; SH, Stonehaven; bold: significant relationship (*P* < 0.05).

#### Translocation factors

The long-term annual mean ESC velocity and its standard deviation were 2.40 ± 0.93 cm s^−1^. No significant relationships were found between the local abundance of *M. atlantica* and interannual variability in the strength of the ESC or the species abundance in the source habitat of the WEC (Table V and Fig. 5).

### Stonehaven: source–sink dynamics at the annual scale

Due to the limited number of observations on the abundance of the eudoxid stage at Stonehaven, only the polygastric stage was considered for this step of the analysis.

A stepwise selection procedure excluded (*α* > 0.05) all of the translocation factors and all of the local environmental factors except food availability (copepod abundance). These exclusions resulted in the analysis amounting to a simple linear regression model of annual *M. atlantica* abundance against food availability. This model explained ~23% of the total variability in the annual abundance of the *M. atlantica* polygastric stage (Table VI).

**Table VI:** Simple linear regression model details for the abundance of the polygastric stage of *Muggiaea atlantica* as a function of food availability (copepod abundance) at Stonehaven from 1999 to 2013

Explanatory variable	$R_{a}^{2}$	d.f.	*F*	*β*
FA	0.23	12	**4.81**	**0.45**

FA, food availability; bold: statistical significance (*P* < 0.05).

## DISCUSSION

The absence of strict physical barriers to the horizontal transport of plankton means that the limits of their spatial distribution are diffuse across wide transitional areas (Beaugrand *et al*., 2002). These limits may shift in space and time in response to seasonal changes in habitat suitability and the hydrographic processes that modulate species translocation (Beaugrand *et al*., 2001). These factors make it challenging to delimit the biogeographic range of plankton species. Whilst previous works (Alvariño, 1971; Mapstone, 2014) defined the latitudinal range of *Muggiaea atlantica* as 59°N–53°S, records of the species’ occurrence this far north in the Atlantic Ocean were exceptional (Totton and Fraser, 1955; Fraser, 1967). Pugh (1999) was more conservative, considering the species’ maximum latitudinal distribution in the Atlantic Ocean as 55°N. In contrast to these earlier findings, our study shows that *M. atlantica* is now a regular constituent of the Scottish coastal plankton (~57–58°N), revealing a dramatic northward expansion of the species’ normal distribution.

Jellyfish populations have an innate propensity to fluctuate in space and time (Lucas and Dawson, 2014). Their opportunistic life history traits, including high fecundity, rapid growth rates and short generation times, enable them to respond to suitable conditions with dramatic localized population increase, a “true” bloom (Graham *et al*., 2001). Conversely, their inability for long-distance volitional swimming means that jellyfish populations are readily dispersed in ocean currents and may aggregate in areas that do not normally support a population, an “apparent” bloom (Graham *et al*., 2001). However, this classification represents opposite ends of a continuum (Graham *et al*., 2001); these biological and physical factors can often combine, resulting in the formation of opportunistic blooms when jellyfish are translocated to areas where suitable conditions exist temporarily (Costello *et al*., 2012). Considering these factors, we investigated the biogeographical expansion of *M. atlantica* in the context of source–sink dynamics (Pulliam, 2000).

We employed several criteria to categorize the two Scottish sampling locations as either source or sink habitats for *M. atlantica*. Indirect evidence of sink habitats has been defined as the absence of reproduction, or insufficient local production to account for recruitment, coupled with the observation of frequent immigration (Pulliam, 2000). We showed that local production accounted for the bulk of polygastric and eudoxid recruitment during the majority of the years studied at both Loch Ewe and Stonehaven. However, during some years local production either did not occur, or occurred in only one of the two life stages, indicating that the species was not always able to complete its full life history, a prerequisite for a source habitat (Pulliam, 2000). The observed patterns of local recruitment therefore reflect features of both source and sink habitats.

Sink habitats are characterized by the absence of the conditions necessary for a species to carry out its full life history (James *et al*., 1984). Temperature has a fundamental influence on animal physiology (Schmidt-Nielsen, 1997) and is a key determinant of reproduction in *Muggiaea* (Purcell, 1982; Carré and Carré, 1991). The temperature optimum for *M. atlantica* identified in our study (~13.0–15.0°C) is in close agreement with results from other areas (Licandro *et al*., 2012; Blackett *et al*., 2014), indicating that the upper limit of the thermal environment at the Scottish sampling locations was periodically suitable for the species to carry out its life cycle. However, in a source habitat, species must be able to exist indefinitely (Pulliam, 2000). As *Muggiaea* are holoplanktonic jellyfish with short-lived larval stages and no known resting stages (Carré and Carré, 1991), other life stages must persist in the water column (Boero *et al*., 2008). Experimental and field observations suggest that overwintering is facilitated by reproductively inactive polygastric stages (Carré and Carré, 1991; Blackett *et al*., 2015). At both Scottish sampling locations, *M. atlantica* exhibited extended periods of absence during the winter and spring. Whilst these absences could simply reflect the species overwintering at an abundance that is below the level of detection of our sampling methodology (Rodriguez-Ramos *et al*., 2013), this was not supported by our observations. In the source habitat of the WEC, *M. atlantica* emerges from overwintering in response to the onset of a critical temperature threshold of 10°C (Blackett *et al*., 2015). Synchronized production with seasonal environmental changes is common in pelagic cnidarians (Purcell *et al*., 2007). If *M. atlantica* was overwintering at the Scottish sampling locations, then it could be expected that local production would commence coincident with a similar temperature threshold. However, this did not occur.

Alternatively, the seasonal periods of absence could represent local annual extinction (Costello *et al*., 2012). Greve (1996) postulated that low winter temperature prevented the establishment of *M. atlantica* in the German Bight. The minimum winter temperatures recorded at Loch Ewe and Stonehaven (6.3°C and 4.9°C, respectively) are lower than observed in the source habitat of the WEC (8.8°C) (Blackett *et al*., 2014), and *M. atlantica* was never recorded when temperatures were below 5.6°C. These low winter temperatures may be below a critical limit for the survival of *M. atlantica*, precluding the existence of an overwintering population. Another possible explanation is that the species is flushed out of the Scottish sampling locations. This scenario could arise if the duration of the non-productive overwintering period exceeds the local water residence times (Costello *et al*., 2012). Blackett *et al*. (2015) suggested that in the WEC, the polygastric stage entered the dormant reproductive stage in response to low temperature in winter (<9.5°C). At both Scottish sampling locations, SST was below this value during ~6 consecutive months of the year (data not shown). Estimates of the surface water residence times of most Scottish lochs and the North Sea Stonehaven sub-region are typically only ~50 days (Rydberg *et al*., 2003; Skogen, 1993), supporting the proposition that *M. atlantica* could be flushed out. If *M. atlantica* cannot overwinter locally, then it must rely on immigration, a defining feature of sink habitats (Pulliam, 2000).

In the absence of direct evidence for immigration, we used the ESC as an index of potential immigration of *M. atlantica* into Scottish coastal waters. Flowing from the Bay of Biscay to the Faroe-Shetland Channel, the ESC is a major section of the poleward flow of warm and saline water in the Northeast Atlantic Ocean (Pingree and Garcia-Soto, 2014; Xu *et al*., 2015). Although the ESC is typically confined to the continental slope, indirect intrusions onto the Scottish Continental Shelf occur *via* periodic flow instabilities and the effects of near surface winds (Inall *et al*., 2009), and direct intrusions regularly occur in winter (Burrows and Thorpe, 1999; Souza *et al*., 2001). Historically, exceptional occurrences of *M. atlantica* in the Scottish Continental Shelf region have been consistently associated with intrusions of the ESC (Fraser, 1967).

Although the ESC influences plankton dispersal in the region (Beaugrand *et al*., 2001; Licandro *et al*., 2010), it also influences the physical environmental conditions of the Scottish Continental Shelf (Inall *et al*., 2009). Therefore, any relationships between the abundance of *M. atlantica* and the ESC may reflect both the direct effects of dispersal on immigration and emigration rates, and the indirect effects of local environmental change on demographic rates. Our variation partitioning analysis allowed us to disentangle the effects of these two processes, providing an estimation of the total, exclusive and combined contributions of local environmental conditions and the ESC on interannual variability in the abundance of *M. atlantica.*

For Loch Ewe, our partial linear regression analysis combining demographic variability (as indexed by SST) and immigration (as indexed by the ESC) explained a large proportion (~62%) of the interannual variation in the abundance of *M. atlantica*. The ESC explained 49%, while SST explained 33% of this total variation, confirming the importance of both the biological and the physical factors. However, these portions of the variation contain a degree of shared variation due to the effect of the ESC on local SST. The partitioning of this variation revealed that the exclusive contribution of SST was minimal, at only 13%. In contrast, the exclusive contribution of the ESC (29%) was a key determinant of the annual abundance of *M. atlantica*. Our results show that interannual variability in the abundance of this species at Loch Ewe is determined by, in order of increasing importance: (i) the availability of suitable local environmental conditions (13%); (ii) the role of the ESC in modulating these environmental conditions (20%); and (iii) the immigration of *M. atlantica* *via* the ESC (29%). In summary, these results indicate that whilst *M. atlantica* is capable of local production during some years, this local production is ultimately dependent upon the arrival of the species *via* immigration.

These results provide compelling indirect evidence that Loch Ewe represents a sink habitat for *M. atlantica*. However, it is necessary to consider other possible explanations. Pseudo-sinks are habitats that are capable of sustaining low-level local production, but where this local production is masked by much higher levels of immigration (Watkinson and Sutherland, 1995). If Loch Ewe was a pseudo-sink habitat, then it could be expected that years with low immigration would still exhibit some low-level population growth. However, this was not observed during several years that were characterized by low flow rates of the ESC. In summary, whilst Loch Ewe may provide a pseudo-sink habitat in some years, periodic local extinctions occur, due to either unsuitable local conditions, or elimination as a result of flushing. These results provide strong support for our classification of Loch Ewe as a true sink habitat.

At Stonehaven, our results are less conclusive. Only food availability had any significant influence on annual abundance, explaining a modest proportion of its interannual variability (23%). Whilst SST was an important determinant at the seasonal scale, its influence on interannual variability was not significant. This could indicate that the relatively low food availability (compared to Loch Ewe) during the seasonal occurrence of *M. atlantica* was an important limiting factor, potentially masking the influence of SST. The lack of any significant relationship with the ESC may be attributable to the relative remoteness of Stonehaven compared with Loch Ewe, which is more directly influenced due to its proximity. At the Fair-Isle Channel the ESC bifurcates, with one stream entering the North Sea along the east coast of Scotland and the other continuing onto the Norwegian shelf (Burrows and Thorpe, 1999). As these latter waters represent a more variable mix of the ESC and Scottish coastal waters compared with Loch Ewe (Inall *et al*., 2009), this could potentially explain the lack of any clear link between *M. atlantica* and the ESC at Stonehaven. Our analysis at the annual scale did not provide any clear evidence on the source–sink status of Stonehaven. However, in light of the patterns of local production and recruitment previously discussed, we tentatively classify it as a sink habitat.

We had hypothesized that occurrence of *M. atlantica* in Scottish coastal waters relied upon translocation from the source habitat of the WEC *via* the ESC. However, our analysis did not show a direct link between the species’ abundance in the WEC and the Scottish sampling locations. However, this does not necessarily refute the existence of any source–sink relationship. Whilst larval dispersal is common in marine planktonic organisms (Cowen and Sponaugle, 2009), *Muggiaea* have short-lived larval stages and no known resting stages (Carré and Carré, 1991). Therefore, the polygastric and eudoxid stages are the most likely dispersal propagules. These life stages may continue active reproduction during translocation, provided that suitable conditions prevail. The ESC travels ~1600 km (Pingree *et al*., 1999) at a mean speed of 10 cm s^−1^ (Xu *et al*., 2015). This equates to an estimated transit time from source to sink of ~6 months. The life cycle of the congeneric *Muggiaea kochi* is in the order of weeks (Carré and Carré, 1991), so the dispersing population could have completed several generations during transit. This process could have the effect of a progressive decoupling of the link between source and sink abundance. Alternatively, the lack of any direct relationship could indicate that a network of different source populations all contribute to the supply of migrant *M. atlantica* arriving in Scottish coastal waters.

We previously demonstrated the establishment of a resident population of *M. atlantica* in the WEC in the 1960s (Blackett *et al*., 2014). Subsequently, an increase in the species’ frequency of occurrence in Irish coastal waters was reported (Jeal and West, 1970). The recent expansion of *M. atlantica* into Scottish coastal waters described in the present study indicates that a progressive expansion of the species’ northern distributional limits has taken place since the 1960s. These results are consistent with published studies that have demonstrated concurrent northward shifts in the biogeography of other species of plankton in the region (Beaugrand *et al*., 2002; Hays *et al*., 2005). The increasing trend in Northern Hemisphere temperature has been demonstrated as an important driver of these changes (Beaugrand *et al*., 2002). Another important factor driving these changes is modification of the path or strength of currents along the European Continental Shelf (Beaugrand *et al*., 2001). Our results highlight the importance of the interplay of these two factors. We propose that the latitudinal range expansion of *M. atlantica* in the northeast Atlantic is the result of increased habitat suitability associated with increasing temperatures, coupled with increased translocation.

Our study has shown that source–sink dynamics can provide a useful framework to understand changes in the distribution of jellyfish species. The effects of global climate change on sea temperature may act to improve habitat suitability in previously inhospitable areas, enabling the formation of new sinks, and the transition of existing sinks to sources. Importantly, our results confirm that current-driven translocation from sources to sinks is an essential factor to consider when investigating changes in species distribution. These factors highlight the importance of improving understanding of the impact of future changes to both sea temperatures and ocean currents. Further research combining field sampling, genetic markers and oceanographic modelling is required to develop our understanding of these processes (Decker *et al*., 2013; Lee *et al*., 2013).

## CONCLUSION

Our study confirms that the distribution of *M. atlantica* has expanded into Scottish coastal waters. Using the concept of source–sink dynamics, we show that this northward expansion involved immigration and the subsequent formation of transient opportunistic populations (sink habitats), and not the establishment of permanent resident populations (source habitats). On the west coast, these changes were attributed to the interplay of two factors: primarily, translocation (as indexed by the ESC) and secondarily, the availability of suitable habitat (as indexed by SST). On the east coast, our results were less conclusive. We hypothesized that immigration into Scottish waters involved direct translocation from the WEC, but our results suggest the existence of a more complex network of sources. In the context of global climate change and its impact on the distribution of jellyfish, these results highlight the importance of the interplay between changes to habitat suitability and current-driven translocation.
